# Postoperative intra-abdominal hypertension predicts worse hospital outcomes in children after cardiac surgery: a pilot study^†^

**DOI:** 10.1093/icvts/ivae019

**Published:** 2024-02-06

**Authors:** Yunyi Zhang, Shuhua Luo, Yuxuan Xie, Yue Wang, Yibing Fang, Shouping Wang, Lijing Deng

**Affiliations:** Department of Anesthesiology, West China Hospital of Sichuan University, Chengdu, 610041, China; Department of Cardiovascular Surgery, West China Hospital of Sichuan University, Chengdu, 610041, China; Department of Anesthesiology, West China Hospital of Sichuan University, Chengdu, 610041, China; Department of Cardiovascular Surgery, West China Hospital of Sichuan University, Chengdu, 610041, China; Department of Cardiovascular Surgery, West China Hospital of Sichuan University, Chengdu, 610041, China; Department of Intensive Care Medicine, West China Hospital of Sichuan University, Chengdu, 610041, China; Department of Intensive Care Medicine, West China Hospital of Sichuan University, Chengdu, 610041, China

**Keywords:** Intra-abdominal hypertension, Cardiac surgery, Paediatrics, Abdominal compartment syndrome, Gastrointestinal complication

## Abstract

**OBJECTIVES:**

Our goal was to determine the incidence and characteristics of postoperative intra-abdominal hypertension (IAH) in paediatric patients undergoing open-heart surgery.

**METHODS:**

This single-centre study included consecutive children (aged <16 years) who underwent open-heart surgery between July 2020 and February 2021. Patients who entered the study were followed until in-hospital death or hospital discharge. The study consisted of 2 parts. Part I was a prospective observational cohort study that was designed to discover the association between exposures and IAH. Postoperative intra-abdominal pressure was measured immediately after admission to the intensive care unit and every 6 h thereafter. Part II was a cross-sectional study to compare the hospital-related adverse outcomes between the IAH and the no-IAH cohorts.

**RESULTS:**

Postoperatively, 24.7% (38/154) of the patients exhibited IAH, whereas 3.9% (6/154) developed abdominal compartment syndrome. The majority (29/38, 76.3%) of IAH cases occurred within the first 24 h in the intensive care unit. Multivariable analysis showed that the Society of Thoracic Surgeons–European Association for Cardio-Thoracic Surgery score [odds ratio (OR) = 1.86, 95% confidence interval (CI) 1.23–2.83, *P* = 0.004], right-sided heart lesion (OR = 5.60, 95% CI 2.34–13.43, *P* < 0.001), redo sternotomy (OR = 4.35, 95% CI 1.64–11.57, *P* = 0.003), high baseline intra-abdominal pressure (OR = 1.43, 95% CI 1.11–1.83, *P* = 0.005), prolonged cardiopulmonary bypass duration (OR = 1.01, 95% CI 1.00–1.01, *P* = 0.005) and deep hypothermic circulatory arrest (OR = 5.14, 95% CI 1.15–22.98, *P* = 0.032) were independent predictors of IAH occurrence. IAH was associated with greater inotropic support (*P* < 0.001), more gastrointestinal complications (*P* = 0.001), sepsis (*P* = 0.003), multiple organ dysfunction syndrome (*P* < 0.001) and prolonged intensive care unit stay (*z* = -4.916, *P* < 0.001) and hospitalization (*z* = -4.710, *P* < 0.001). The occurrence of a composite outcome (*P* = 0.009) was significantly increased in patients with IAH.

**CONCLUSIONS:**

IAH is common in children undergoing cardiac surgery and is associated with worse hospital outcomes. Several factors may be associated with the development of IAH, including basic cardiac physiology and perioperative factors.

**Trial information:**

This study was registered in the Chinese Clinical Trial Registry (Trial number: ChiCTR2000034322)

URL site: https://www.chictr.org.cn/hvshowproject.html?id=41363&v=1.4

**Figure:**
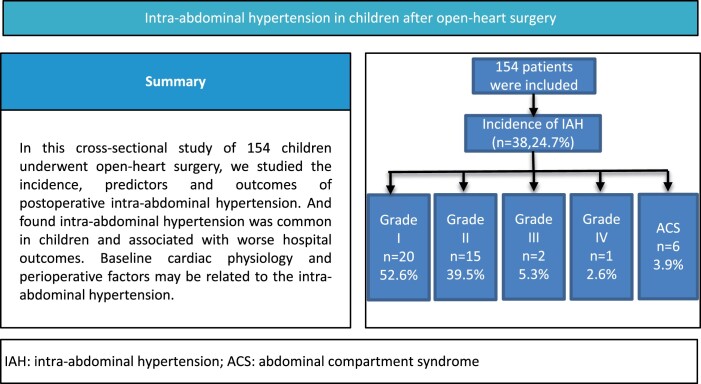

## INTRODUCTION

The postoperative occurrence of intra-abdominal hypertension (IAH) is frequent in adult cardiac surgery, with incidence rates ranging from 27% to 83% [1–6]. Evidence suggests that increased intra-abdominal pressure (IAP) has an adverse effect on cardiac output, splanchnic blood flow and breathing mechanics, leading to postoperative organ dysfunction [1–6]. Therefore, routine postoperative IAP measurement is recommended in high-risk adult patients undergoing cardiac surgery to prevent the deleterious effects of IAH.

A recent prospective epidemiological study that used the updated World Society of Abdominal Compartment Syndrome (WSACS) guidelines [7] showed that IAH is associated with higher mortality [8] and organ dysfunction, even at lower levels in children [9]. Paediatric patients undergoing open-heart surgery may be prone to developing postoperative IAH due to the various aspects of cardiopulmonary bypass (CPB) that can potentially predispose children to IAH, such as an inflammatory response, capillary leakage and splanchnic hypoperfusion [9]. However, the incidence of IAH in this population is poorly understood.

The primary goal of this study was to determine the incidence and characteristics of postoperative IAH in paediatric patients undergoing open-heart surgery. We then examined the predictors of IAH and its impact on the occurrence of hospital-related adverse outcomes.

## PATIENTS AND METHODS

### Ethics statement

This study was registered in the Chinese Clinical Trial Registry (ChiCTR2000034322). Approval was obtained from the research ethics board of the West China Hospital (2020, No.547), and informed consent for the paediatric participants was obtained from their parents/guardians.

### Study population

This single-centre study included consecutive children (aged <16 years) who underwent on-pump cardiac surgery between July 2020 and February 2021. Patients with univentricular physiology or those unsuitable for urine catheter placement were excluded. Patients who entered the study were followed until in-hospital death or hospital discharge. In-hospital death included an encounter with a discharge status of died or died in a medical facility. This study had 2 parts. Part I was a prospective observational cohort study that was designed to discover the association between exposures and IAH. Part II was a cross-sectional study to compare the hospital-related adverse outcomes between the IAH and the no-IAH cohorts. Patients were divided into 3 categories based on their diagnosis and physiology (Supplemental Table 1).

Applying the “1 in 10” rule to estimate the sample size for logistic regression, at least 10 cases per covariate were needed in the minority class. Because we had a sample of 154 people in this study, of which 38 developed IAH, we were able to fit 3 variables reliably in the multivariable regression model.

### Intra-abdominal pressure measurement

Bladder pressure was measured through a Foley bladder catheter using the modified Kron technique, a method endorsed by consensus guidelines [7]. An appropriate volume of sterile saline (1 ml/kg, minimum optimal volume 3 ml, maximum volume 25 ml) was instilled into the bladder. The IAP was measured using a pressure transducer calibrated to the level of the mid-axillary line and expressed in mmHg. All nasogastric tubes were opened, and the patients were not paralysed for IAP pressure measurements; however, measurements were taken with the patient in a completely supine position with adequate sedation. Because infants have a faster respiratory rate than adults, the acquisition of measurements at end expiration was challenging. Thus, the IAP in our study was recorded after approximately 1 min to stabilize the count.

Baseline IAP measurements were performed after the induction of anaesthesia in all patients. Postoperative IAP was routinely measured immediately after the patient was admitted to the intensive care unit (ICU) and every 6 h thereafter until the Foley catheter was removed, abdominal drainage, peritoneal dialysis or in-hospital death, whichever occurred first. IAH was defined as a sustained or repeated pathological elevation in IAP>10 mmHg. Also, the classifications of IAH and abdominal compartment syndrome (ACS) were defined in accordance with the WSACS using the proposed specific diagnostic criteria for infants and children [7] (Supplemental Table 2).

### Cannulation strategy

Extracorporeal circulation was established via the ascending aorta and the superior and inferior venae cavae. If reconstruction of the aortic arch was required, an arterial cannula was inserted into the innominate artery using a prosthetic vessel with an end-to-end anastomosis for selective perfusion of the upper body or brain while an arterial cannula was placed in the descending aorta for perfusion of the lower body.

### Management in the intensive care unit

Patients were ventilated postoperatively using pressure-controlled ventilation (tidal volume 6–10 ml/kg, peak inspiratory pressure <30 cm H_2_O, respiratory rate 10–30 breaths/min), with adjustments to maintain normocapnia. Haemodynamics were maintained via fluids or vasoactive drugs based on central venous pressure (CVP), mean atrial blood pressure, lactate levels and central venous saturation. Extubation was performed once the patient was awake, haemodynamically stable and within an acceptable oxygen saturation range after blood gas analysis. Transthoracic echocardiography was performed on postoperative day 1 to assess the adequacy of surgical repair and ventricular function.

Nasogastric tubes were routinely placed upon admission of the patient to the ICU. Enteral nutrition usually began within 6 h of admission unless the patient was haemodynamically unstable or extubation was anticipated. An initial trophic feed rate of 30–100 kcal/kg/day was used, and human milk was preferred for infants. Feeding was advanced when tolerated, and intermittent feeding was used whenever possible. Gastrointestinal (GI) complications were monitored and recorded by bedside nurses based on the definitions of abdominal complications published by the Multi-Societal Database Committee for Paediatric and Congenital Heart Disease [10].

### Data collection

The mechanism underlying IAH in paediatric patients after cardiac surgery remains unknown. Accordingly, in this study, all factors suspected to influence the probability of the occurrence of IAH in cardiac surgery according to previous studies were compiled [6]. Exposures of IAH included the evaluation of demographic data, liver and kidney function, cause of admission, the Society of Thoracic Surgeons–European Association for Cardio-thoracic Surgery (STAT) score and intraoperative intervention. The primary outcomes were the occurrences of IAH and ACS in children who underwent open-heart surgery. Secondary outcomes included composite morbidity–mortality outcomes (Supplemental Table 3), maximal vasoactive-inotropic score calculated every 24 h of stay in the ICU, GI complications, sepsis, multiple organ dysfunction syndrome (MODS), duration of mechanical ventilation, length of stay in the ICU and length of hospitalization [11–13].

### Statistical analyses

Non-continuous variables are expressed as medians and quartiles or as absolute numbers with percentages. The Mann–Whitney U test was used to compare continuous non-normal variables, whereas the Student *t*-test was employed to compare continuous variables. The paired Wilcoxon signed-rank test was used to compare the baseline IAP and the IAP immediately after admission to the ICU. Univariate and multivariable logistic regression analyses were used to identify the association between the exposures and IAH. Multivariable analysis results were summarized by estimating the odds ratios (OR) and their respective 95% confidence intervals (CI). The receiver operating characteristic (ROC) curve and area under the curve (AUC) were used to estimate the accuracy of continuous variables in the final regression model. We also used restricted cubic splines with 3 knots at the 10th, 50th and 90th to flexibly model the associations between CPB duration and IAH. In all comparisons, a *P*-value of <0.05 was considered statistically significant. Data were analysed using Stata/SE version 17.0 (StataCorp LP, College Station, TX, USA).

## RESULTS

### Demographics

Patient demographic information is summarized in Table 1. In total, 154 consecutive patients were enrolled in this study. The median age and weight were 25 (8.4,69.9) months and 11 (7,19.5) kg, respectively. Approximately one-fifth (22.1%) of all patients had a right-sided heart lesion (34/154).

**Table 1:** Baseline clinical characteristics of patients.

Variables	Total (n = 154)	IAH (n = 38)	No IAH (n = 116)	*z*	*P-*value
Male, n (%)	80 (52)	15 (39.5)	59 (50.9)		0.223
Preterm birth, n (%)	4 (2.6)	4 (10.5)	0 (0)		<0.001
^#^Age (month)	25 (8.4,69.9)	13.8 (5.7,55.4)	26.9 (9.2,74.65)	1.666	0.096
^#^Weight (kg)	11 (7,19.5)	8.25 (7,14)	12 (7,20)	1.727	0.084
^#^BMI (kg/m^2^)	15.6 (14.2,16.8)	16 (14.9,17.3)	15.6 (14.1,16.8)	−1.134	0.258
^#^Preoperative ALT (IU/l)	16 (11,21)	16 (11,30)	16 (12,21)	−0.315	0.755
^#^Preoperative AST (IU/l)	34 (27,43)	34.5 (25,47)	34 (27,41)	−0.444	0.659
^#^Preoperative sCr (μmol/l)	30.5 (26,40)	29 (24,36)	31.5 (27,40)	1.789	0.074
STAT score, n (%)					0.003
1	64 (41.6)	6 (15.8)	58 (50)		
2	61 (39.6)	22 (57.9)	39 (33.6)		
3	19 (12.3)	6 (15.8)	13 (11.2)		
4	10 (6.5)	4 (10.5)	6 (5.2)		
Right-sided heart lesion, n (%)	34 (22.1)	16 (42.1)	18 (15.5)		<0.001
Left-sided heart lesion, n (%)	23 (14.9)	7 (18.4)	16 (13.8)		<0.001
Others, n (%)	97 (63)	15 (39.5)	82 (70.7)		0.001
Emergency surgery, n (%)	4 (2.6)	1 (2.6)	3 (2.6)		0.988
Redo sternotomy, n (%)	23 (14.9)	11 (28.9)	12 (10.3)		0.005
^#^Baseline CVP (mmHg)	10 (7.3,12.2)	11.2 (9.3,13.3)	9.2 (7,12)	−2.748	0.005
^#^Baseline IAP (mmHg)	3 (2,4)	4 (3,5)	3 (2,4)	−2.985	0.003
^#^CPB duration (min)	110 (76,145)	130.5 (109,198)	99 (73,137)	−3.726	<0.001
Deep hypothermic circulatory arrest, n (%)	8 (5.2)	5 (13.2)	3 (2.6)		0.01
^#^Fluid balance (ml)	100 (35,220)	87.5 (20,235)	105 (60,200)	0.526	0.602
^#^Transfusion needs					
RBC (U)	1.5 (1,1.5)	1.5 (1,2)	1 (1,1.5)	−1.277	0.242
Autologous blood (ml)	150 (100,300)	160 (100,344)	150 (100,200)	−0.227	0.823
Plasma (ml)	100 (60,200)	100 (100,200)	80 (20,200)	−0.925	0.374
PLT (U)	1 (1,1)	1 (1,1)	0.75 (0.5,1)	−2.159	0.080
^#^Haematocrit (%)					
Baseline	35 (32,39)	36 (36,32,42)	35 (33,37)	−1.020	0.310
During CPB	24 (21,26)	23 (20,26)	24 (21,26)	0.151	0.882
At the end of the operation	27 (25,29)	27 (24,29)	26 (25,28)	−0.466	0.644

# Non-continuous variables are expressed as median and quartile.

ALT: alanine aminotransferase; AST: aspartate aminotransferase; BMI: body mass index; CPB: cardiopulmonary bypass; CVP: central venous pressure; IAH: intra-abdominal hypertension; IAP: intra-abdominal pressure; PLT: platelet; RBC: red blood cells; sCr: serum creatinine; STAT: Society of Thoracic Surgeons–European Association for Cardio-thoracic Surgery.

### Occurrence of postoperative intra-abdominal hypertension and abdominal compartment syndrome

**Figure 1:**
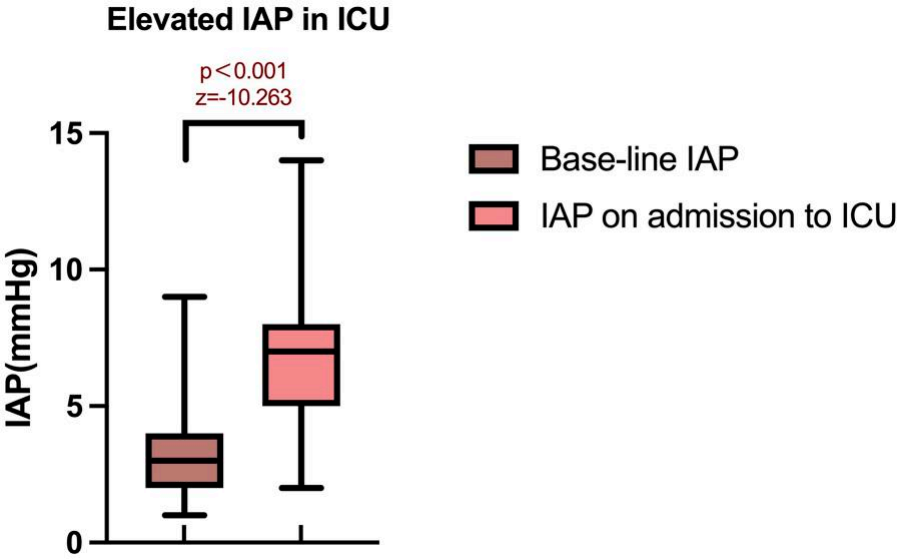
The intra-abdominal hypertension on admission to the intensive care unit was significantly higher than at baseline (*P* < 0.001). IAP: intra-abdominal pressure; ICU: intensive care unit.

**Figure 2:**
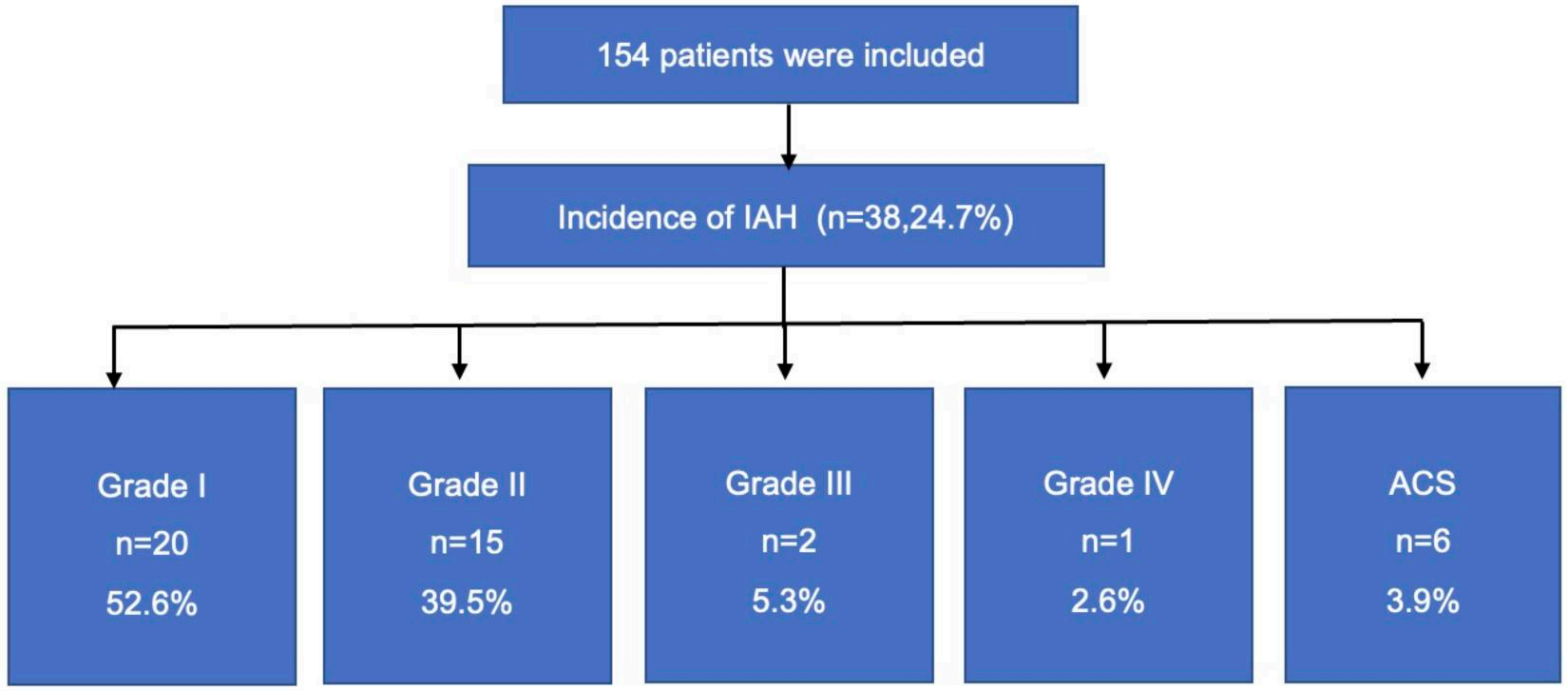
In the final analysis, 38 (24.7%) patients developed intra-abdominal hypertension postoperatively. Twenty patients had grade I intra-abdominal hypertension (IAH); 15 had grade II IAH; 2 had grade III IAH; and 1 had grade IV IAH. Six patients (3.9%) developed abdominal compartment syndrome. ACS: abdominal compartment syndrome; IAH: intra-abdominal hypertension.

A total of 1745 IAP measurements were performed. The IAP on admission to the ICU [7(5,8) mmHg] was significantly higher than that at baseline [3(2,4) mmHg, *z* = −10.263, *P* < 0.001] (Fig. 1). Approximately one-fourth (24.7%) of the patients (38, 38/154) developed IAH during their stay in the ICU, whereas no patient had IAH at baseline. The majority (35, 35/38) of patients with IAH had grades I (*n* = 20) and II (*n* = 15) IAH, and the remaining 3 patients had grades III (*n* = 2) and IV (*n* = 1) IAH (Fig. 2). Six patients (6/154, 3.9%) were diagnosed with ACS, yet no patient required abdominal surgery after consultation with the general surgical team. In patients with IAH (*n* = 38), the majority (29/38,76.3%) occurred within the first 24 h in the ICU.

### Predictors of intra-abdominal hypertension development

**Figure 3:**
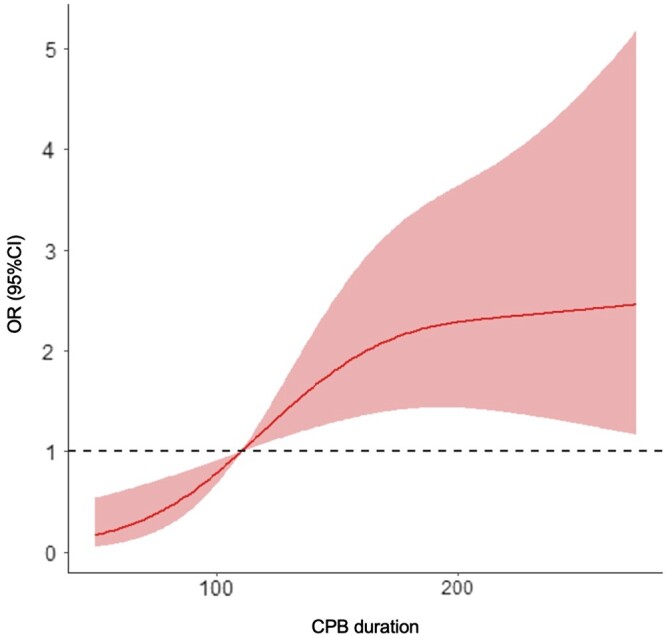
Restricted cubic spline curves of associations between cardiopulmonary bypass duration and intra-abdominal hypertension. Restricted cubic spline regression models were conducted with 3 knots at the 10th, 50th and 90th initial cardiopulmonary bypass durations. The red lines represent the 95% confidence intervals for the spline model. CI: confidence interval; CPB: cardiopulmonary bypass; OR: odds ratio.

Univariate analysis showed that IAH was associated with the STAT score (OR = 1.84, 95% CI 1.22–2.77, *P* = 0.004), redo sternotomy (OR = 3.50, 95% CI 1.41–8.87, *P* = 0.007), high baseline IAP (OR = 1.43, 95% CI 1.13–1.82, *P* = 0.003), right-sided heart lesion (OR = 5.12, 95% CI 2.20–11.96, *P* < 0.001), high baseline CVP (OR = 1.19, 95% CI 1.06–1.33, *P* = 0.002), prolonged CPB duration (OR = 1.01, 95% CI 1.00–1.01, *P* = 0.004) and deep hypothermic circulatory arrest (DHCA) (OR = 5.71, 95% CI 1.30–25.15, *P* = 0.002). In the multivariable analysis, when adjusted for age and sex, the study reflects that STAT scores (OR = 1.86, 95% 1.23–2.83, *P* = 0.004), a right-sided heart lesion (OR = 5.60, 95% CI 2.34–13.43, *P* < 0.001), redo sternotomy (OR = 4.35, 95% CI 1.64–11.57, *P* = 0.003), high baseline intra-abdominal pressure (OR = 1.43, 95% CI 1.11–1.83, *P* = 0.005), prolonged CPB duration (OR = 1.01, 95% CI 1.00–1.01, *P* = 0.005) and deep hypothermic circulatory arrest (OR = 5.14, 95% 1.15–22.98, *P* = 0.032) were linked to IAH (Table 2). Furthermore, the restricted cubic spline curve indicated that the risk of IAH may increase with the delay in the initial CPB duration (Fig. 3).

**Table 2:** Predictors of intra-abdominal hypertension.

	Univariable analysis			Multivariable analysis			*P*-value for difference between AUCs (95% CI)
	OR	95% CI	*P*-value	OR	95% CI	*P*-value	
Age (months)	0.99	0.99–1.00	0.204				
Sex	0.63	0.30–1.33	0.225				
Preoperative sCr (μmol/l)	0.96	0.93–1.00	0.075				
*STAT score	1.84	1.22–2.77	0.004	1.86	1.23–2.83	0.004	
*Right-sided heart lesion (n)	5.12	2.20–11.96	<0.001	5.60	2.34–13.43	<0.001	
*Redo sternotomy (n)	3.50	1.41–8.87	0.007	4.35	1.64–11.57	0.003	
*Baseline CVP (mmHg)	1.19	1.06–1.33	0.002	1.20	1.07–1.35	0.001	0.004
							(0.55–0.75)
*Baseline IAP (mmHg)	1.43	1.13–1.82	0.003	1.43	1.11–1.83	0.005	0.003
							(0.56–0.76)
*CPB duration (min)	1.01	1.00–1.01	0.004	1.01	1.00–1.01	0.005	＜0.001
							(0.61–0.79)
*Deep hypothermic circulatory arrest (n)	5.71	1.30–25.15	0.021	5.14	1.15–22.98	0.032	

* Adjusted for age and sex in the multivariable analysis.

AUC: area under the curve; CI: confidence interval; CPB: cardiopulmonary bypass; CVP: central venous pressure; GI: gastrointestinal; IAP: intra-abdominal pressure; sCr: serum creatinine; STAT: Society of Thoracic Surgeons–European Association for Cardio-thoracic Surgery.

**Figure 4:**
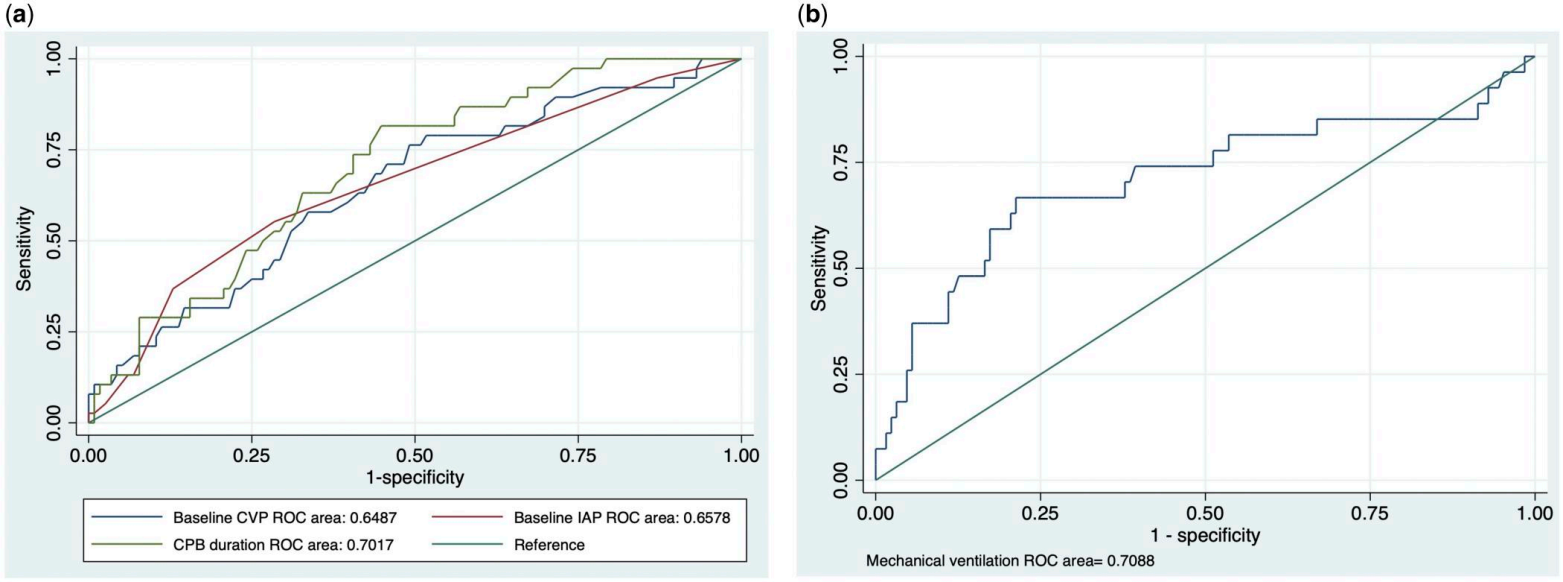
(**a**) Receiver operating characteristic curve and area under the curve of predictors and intra-abdominal hypertension; (**b**) receiver operating characteristic curve and area under the curve of risk factors and composite outcomes. CVP, central venous pressure; IAP, intra-abdominal pressure; ROC, receiver operating characteristic.

In the “predictors of IAH” logistic model, ROC characteristics showed a baseline CVP AUC value of 0.6487 (95% CI 0.55–0.75, *P* = 0.004), baseline IAP AUC of 0.6578 (95% CI 0.56–0.76, *P* = 0.003) and CPB duration AUC of 0.7017 (95% CI 0.61–0.79, *P* < 0.001) (Fig. 4a, Table 2).

### Intra-abdominal hypertension and adverse hospital outcomes

Three in-hospital deaths occurred, corresponding to a mortality rate of 1.9%. Two patients had irreversible cardiac dysfunction, and 1 died of a severe pulmonary infection. Patients with IAH were more likely to have greater inotropic support at 24, 48 and 72 h (*P* < 0.001) and longer stays in the ICU (z = −4.916, *P* < 0.001) and in the hospital (*z* = −4.710, *P* < 0.001). Furthermore, major complications such as sepsis (*P* = 0.003), MODS (*P* < 0.001), GI complications (*P* = 0.001) and the composite outcome (*P* = 0.009) also occurred more frequently in patients with IAH (Table 3).

**Table 3:** Hospital outcomes between the intra-abdominal hypertension and the no-intra-abdominal hypertension cohorts.

	Total (n = 154)	IAH (n = 38)	No-IAH (n = 116)	*z*	*P*-value
^#^Mechanical ventilation time (h)	16.8 (8.6, 81.2)	89.6 (27.5,218.8)	12.3 (7.9,31.4)	−4.652	<0.001
^#^VIS (24 h)	6.8 (2,12.5)	12.8 (8.20.4)	5.3 (0,10)	−4.779	<0.001
^#^VIS (48 h)	6 (0,11)	11 (8,17)	5 (0,8.2)	−5.222	<0.001
^#^VIS (72 h)	5 (0,10.2)	10.4 (7,12.9)	0 (0,7.3)	−4.908	<0.001
GI complications, n (%)	10 (6.5)	7 (18.4)	3 (2.6)		0.001
Sepsis, n (%)	9 (5.8)	6 (15.8)	3 (2.6)		0.003
MODS, n (%)	14 (9.1)	9 (23.7)	5 (4.3)		<0.001
Composite outcome, n (%)	27 (17.5)	12 (31.6)	15 (12.9)		0.009
AKI, n (%)	4 (2.6)	2 (5.3)	2 (1.7)		
Liver failure, n (%)	12 (7.8)	7 (18.4)	5 (4.3)		
Lactic acidosis, n (%)	17 (11)	7 (18.4)	10 (8.6)		
In-hospital deaths, n (%)	3 (1.9)	1 (2.6)	2 (1.7)		
Cardiac arrest, n (%)	5 (3.2)	1 (2.6)	4 (3.4)		
ECMO, n (%)	2 (1.3)	0 (0)	2 (1.7)		
AKI, n (%)	4 (2.6)	2 (5.3)	2 (1.7)		
^#^ICU duration (days)	4 (2,8)	8 (6,16)	3 (2,6.5)	−4.916	<0.001
^#^Hospital duration (days)	7 (6,13)	11.5 (9,19)	6 (5,10)	−4.710	<0.001

# Non-continuous variables are expressed as median and quartile.

AKI: acute kidney injury; ECMO; extracorporeal membrane oxygenation; GI: gastrointestinal; IAH: intra-abdominal hypertension; ICU: intensive care unit; MODS; multiple organ dysfunction syndrome; VIS: vasoactive-inotropic score.

The univariate analysis demonstrated that the duration of mechanical ventilation (OR = 1.00, 95% CI 1.00–1.01, *P* = 0.001), sepsis (OR = 21.88, 95% CI 4.24–112.86, *P* < 0.001) and IAH (OR = 3.11, 95% CI 1.30–7.44, *P* = 0.011) were risk factors for composite outcomes. Similarly, IAH (OR = 3.60, 95% CI 1.45–8.94, *P* = 0.006), mechanical ventilation duration (OR = 1.01, 95% CI 1.00–1.01, *P* = 0.001) and sepsis (OR = 27.65, 95% CI 4.99–153.25, *P* < 0.001) were identified as independent risk factors in the multivariable analysis (Table 4).

**Table 4:** Risk factors of composite outcomes.

	Univariable analysis			Multivariable analysis			*P*-value for difference between AUCs (95% CI)
	OR	95% CI	*P*-value	OR	95% CI	*P*-value	
Age (months)	1.00	1.00–1.01	0.607				
Sex	1.73	0.74–4.02	0.203				
*Mechanical ventilation (h)	1.00	1.00–1.01	0.001	1.01	1.00–1.01	0.001	0.001
							(0.58–0.84)
*Postoperative RV dysfunction(n)	0.30	0.11–0.84	0.021				
*Postoperative LV dysfunction (n)	2.31	0.80–6.68	0.124				
*Sepsis (n)	21.88	4.24–112.86	<0.001	27.65	4.99–153.25	<0.001	
*IAH (n)	3.11	1.30–7.44	0.011	3.60	1.45–8.94	0.006	

* Adjusted for age and sex in the multivariable analysis.

AUC: area under the curve; CI: confidence interval; IAH: intra-abdominal hypertension; LV: left ventricle; OR: odds ratio; RV: right ventricle.

In the “risk factors of composite outcomes” logistic model, ROC characteristics showed a mechanical ventilation AUC value of 0.7088 (95% CI 0.58–0.84, *P* = 0.001) (Fig. 4b, Table 4).

## DISCUSSION

Despite the recent surge in interest in IAH in critically ill children, data on IAH in paediatric patients undergoing cardiac surgery are scarce. The goal of this study was to describe the clinical characteristics of IAH in children who underwent open-heart surgery. The outcome of our investigation revealed that 24.7% of paediatric patients who underwent cardiac surgery experienced IAH. Prolonged CPB duration and DHCA, independently preceding the occurrence of IAH, suggest that the inflammation provoked by CPB may be a crucial factor in IAH development. Patients with IAH have various adverse hospital outcomes, highlighting the importance of increasing awareness of this situation among critical care physicians in the paediatric cardiac ICU. However, it is as yet unclear if IAH is a marker of increased critical illness severity or a condition per se. The question remains as to whether the prevention or treatment of IAH improves clinical outcomes. Future efforts should focus on defining predictors of IAH development in a larger cohort and identifying whether interventions aimed at reducing IAP lead to fewer patient deaths.

### Incidence and characteristics of intra-abdominal hypertension

The incidence of IAH and ACS in the current study was comparable to that in previous reports of critically ill children. However, it was seen less frequently in children than in adult patients after cardiac surgery, with incidence rates ranging from 26.9%–83.3%, according to several studies [1–6, 8–9, 14]. This result may be linked to better abdominal wall compliance in children [8]. Abdominal compliance is a dynamic variable expressed as the change in the intra-abdominal volume per change in the intra-abdominal pressure [15]. Blaser *et al.* reported that abdominal compliance could decrease in the elderly owing to the reduced elasticity of the abdominal wall [15]. Medical conditions such as chronic obstructive pulmonary disease, hypertension and aortic atheroma also contribute to decreased abdominal compliance in adult patients [2, 15]. In contrast, children can distend their abdomen in response to increasing intra-abdominal volume, resulting in a lower IAP.

In line with previous studies, the majority of IAH cases occurred early after ICU admission, post open-heart surgery [2, 4, 5, 14, 16]. This result underscores the importance of conducting IAP measurements within 24 h after surgery, particularly in high-risk populations. The WSACS medical management algorithm proposes 5 treatment options for nonsurgical IAH management: (i) evacuation of intraluminal contents, (ii) evacuation of intra-abdominal space-occupying lesions, (iii) improvement of abdominal wall compliance, (iv) optimization of fluid administration and (v) optimization of systemic and regional perfusion [7]. Once IAH occurs in children, standardized protocols should be implemented immediately to prevent further organ dysfunction and avoid progression to ACS.

### Predictors of intra-abdominal hypertension

Consistent with the limited literature on adult cardiac patients, we were able to discover the deleterious effects of CPB on IAP in paediatric patients [6, 17–18]. CPB produces a generalized and vigorous inflammatory response that, when associated with splanchnic ischaemia-reperfusion, may compromise the bowel capillary endothelium, leading to increased microvascular permeability and gut oedema [17]. DHCA was also identified as an independent predictor of IAH. This result could be attributed to the fact that DHCA involves multiple ischaemic vascular territories with a pronounced inflammatory response during reperfusion. The similarities among these predictors further highlight the pathogenetic and pathophysiological similarities of IAH/ACS between adults and children. This observation enhances the probability that these risk factors are perceived as potential evidence-based risk indicators for both adults and children until they are formally evaluated in a prospective multicentre observational study in these 2 patient populations.

In addition to the afore-mentioned predictors, the current study showed that right-sided heart disease independently predicted the occurrence of IAH. High preoperative CVP and postoperative right ventricular (RV) dysfunction are likely to contribute to the development of IAH. RV dysfunction is associated with low cardiac output and is characterized by elevated CVP [16]. Subsequent splanchnic venous stasis and gastrointestinal oedema may increase the intra-abdominal volume and lead to IAH. The increased IAP, on the other hand, could further compromise RV function by raising the pleural pressure, increasing pulmonary vascular resistance and elevating the diaphragm. Treatments that could decrease IAP, such as adequate sedation and negative fluid balance, are likely to break through the vicious cycle in patients with RV dysfunction [2].

### Intra-abdominal hypertension and adverse hospital outcomes

In line with previous articles in the literature, IAH was associated with a high incidence of postoperative complications, such as sepsis and MODS, in the current cohort [2, 8, 16, 19].

Recently, GI complications have been associated with prolonged hospital stays and increased mortality after cardiac surgery in children [20]. A compromised splanchnic blood supply caused by low cardiac output was considered the primary reason for GI complications. Such an outcome indicates that IAH may play an important role in the development of GI complications. We hypothesised that increased IAP could further exacerbate abdominal organ ischaemia by decreasing the abdominal perfusion pressure. Evidence from prior investigations show that IAH has a detrimental effect on organ blood flow [2, 4, 8]. However, further studies are warranted to elucidate the mechanisms underlying the development of GI complications after cardiac surgery.

We further identified IAH as an independent risk factor associated with adverse hospital composite outcomes. The difference in the composite outcomes seems to be driven mainly by liver failure and lactic acidosis. Liver failure can be ascribed to severely impaired hepatic vascular flow due to elevated IAP [21]. Increased lactate production results from impaired oxygen delivery, which is caused mainly by decreased cardiac output [2]. Several mechanisms such as direct compression of the heart, decreased contractility due to displacement of the diaphragm and decreased venous return due to compression of the inferior vena cava have been suggested to decrease cardiac output in the presence of IAH [22]. The consequent liver dysfunction can compromise lactate clearance and exacerbate lactic acidosis. However, the incidences of in-hospital death, circulatory support and renal insufficiency were not statistically significant in the composite outcomes, which may be due to the relatively small sample size. Despite the increased morbidity and mortality associated with IAH/ACS, it remains unclear whether the prevention or treatment (either surgical or medical) of IAH/ACS among critically ill patients improves patient outcomes. Therefore, some researchers have questioned whether these conditions are simply markers of an increased severity of critical illness. Indeed, a high disease severity score was significantly associated with the development of IAH and ACS in paediatric and adult studies [1, 4]. With our current understanding of the pathophysiology and epidemiology of IAH/ACS, future efforts in paediatric studies should focus on defining evidence-based risk factors of IAH and compartment syndrome development and determining whether interventions aimed at reducing IAP will lead to fewer patient deaths.

### Limitations

This study has several limitations. First, in-hospital death, circulatory support and renal insufficiency were rare in our study, probably due to the relatively small sample size. Secondly, IAP was not used to account for analgesia, sedation and neuromuscular blockers in our study. Third, abdominal breathing in children with respiratory distress may have resulted in falsely high IAP readings due to abdominal muscle contractions. This confounding factor can be eliminated by adequate sedation and/or neuromuscular blockade in mechanically ventilated children. Fourth, patients in our study were not recruited based on target sample size, but rather there were 154 patients available for this study and, based on the “1 in 10” rule, we decided to create a model of 3 variables to avoid overfitting. Fifth, only statistical criteria, not theoretical arguments, were used to include variables in our cohort models.

## CONCLUSION

IAH is common in children undergoing cardiac surgery and is associated with worse hospital outcomes. Several factors, including basic cardiac physiology and intraoperative factors, may be associated with the development of IAH.

## Supplementary Material

ivae019_Supplementary_Data

## Data Availability

The data underlying this article will be shared on reasonable request to the corresponding author.

## ABBREVIATIONS

ACS: Abdominal compartment syndrome
AUC: Area under the curve
CPB: Cardiopulmonary bypass
CVP: Central venous pressure
DHCA: Deep hypothermic circulatory arrest
GI: Gastrointestinal
IAH: Intra-abdominal hypertension
IAP: Intra-abdominal pressure
ICU: Intensive care unit
MODS: Multiple organ dysfunction syndrome
ROC: Receiver operating characteristic
WSACS: World Society of Abdominal Compartment Syndrome
