# Supplementary material for: Postoperative intra-abdominal hypertension predicts worse hospital outcomes in children after cardiac surgery: a pilot study
Source: Interdiscip Cardiovasc Thorac Surg. 2024 Feb 6;38(2):ivae019. doi: 10.1093/icvts/ivae019 (PMC10882438; doi:10.1093/icvts/ivae019)
Supplement: ivae019_Supplementary_Data [file ivae019_supplementary_data.docx]

| Categories | Right-sided heart lesion | Left-sided heart leision | Others |
| --- | --- | --- | --- |
| Intra-operative diagnosis | Tetralogy of Fallot (TOF)  Pulmonary artery stenosis  Pulmonary valve absents  Pulmonary atresia with ventricular septal defect  Right ventricular outflow tract obstruction (RVOTO) Tricuspid regurgitation (TR) | Mitral stenosis  Mitral regurgitation  Mitral prolapse  Aortic stenosis  Aortic insufficiency  Aortic subvalvular septum  Aortic bicuspid malformation  Aortic coarctation  Left ventricular outflow tract obstruction | Coronary artery anomaly  Ventricular septal defect(VSD)  Atrial septal defect(ASD) |

Supplemental Table 1: Categorie and diagnosis of patients

Supplemental Table 2: Proposed pediatric specific definitions by the WSACS

| Terminlogogy | Proposed definition in children |
| --- | --- |
| IAH | A sustained or repeated pathological elevation in IAP＞10 mmHg |
| IAH grade I | IAP 10–12 mmHg |
| IAH grade II | IAP 13–15 mmHg |
| IAH grade III | IAP 16–19 mmHg |
| IAH grade IV | IAP ≥20 mmHg |
| ACS | A sustained elevation in IAP＞10 mmHg associated with new or worsening organ dysfunction that can be attributed to elevated IAP |

WSACS: The World Society of the Abdominal Compartment Syndrome; IAH:intra-abdominal hypertension; ACS: abdominal compartment syndrome; IAP:intra-abdominal pressure

Supplemental Table 3: Definition of composite outcome

| Composite outcome | Definition |
| --- | --- |
| AKI | An increase in creatinine during admission greater than 2 times the upper limit of normal (>1.5 mg/dL) |
| Liver failure | Aspartate aminotransferase or alanine aminotransferase greater than 2 times the upper limit of normal (AST >80 IU/L; ALT >80 IU/L). |
| Lactic acidosis | An increasing arterial lactate concentration that reached more than 5 mmol/dL postoperatively |
| Death | Irreversible cessation of circulatory and respiratory functions, or irreversible cessation of all functions of the entire brain, including the brain stem |
| Cardiac arrest | Circulatory collapse requiring cardiopulmonary resuscitation |
| ECMO | Need extracorporeal membrane oxygenation support postoperatively |

AKI: acute kidney injury; ALT: alanine aminotransferase; AST: aspartate aminotransferase; ECMO: extracorporeal membrane oxygenation

~~Supplemental Table 4: Risk factors of composite outcome~~

|  | ~~Univariate analysis~~ | | | ~~Multivariate analysis~~ | | |
| --- | --- | --- | --- | --- | --- | --- |
|  | ~~OR~~ | ~~95%CI~~ | ~~P-value~~ | ~~OR~~ | ~~95%CI~~ | ~~P-value~~ |
| ~~Age(months)~~ | ~~1.00~~ | ~~-0.00-0.00~~ | ~~0.72~~ |  |  |  |
| ~~Weight(kg)~~ | ~~1.01~~ | ~~0.98-1.04~~ | ~~0.60~~ |  |  |  |
| ~~BMI(kg/m~~^~~2~~^~~)~~ | ~~0.98~~ | ~~0.89-1.09~~ | ~~0.73~~ |  |  |  |
| ~~Preoperative ALT(IU/L)~~ | ~~1.00~~ | ~~0.98-1.02~~ | ~~0.90~~ |  |  |  |
| ~~Preoperative AST(IU/L)~~ | ~~0.99~~ | ~~0.97-1.01~~ | ~~0.62~~ |  |  |  |
| ~~Preoperative Scr (umol/L)~~ | ~~1.02~~ | ~~0.99-1.06~~ | ~~0.12~~ |  |  |  |
| ~~Emergency surgery(n)~~ | ~~4.55~~ | ~~0.61-33.79~~ | ~~0.14~~ |  |  |  |
| ~~Mechanical ventilation(hours)~~ | ~~1.1~~ | ~~1.06-1.23~~ | ~~0.001~~ | ~~1.1~~ | ~~1.01-1.20~~ | ~~0.03~~ |
| ~~Postoperative RV dysfunction(n)~~ | ~~0.4~~ | ~~0.17-1.09~~ | ~~0.07~~ | ~~0.4~~ | ~~0.12-1.05~~ | ~~0.06~~ |
| ~~Postoperative LV dysfunction(n)~~ | ~~2.07~~ | ~~0.72-5.95~~ | ~~0.18~~ |  |  |  |
| ~~Sepsis(n)~~ | ~~19.6~~ | ~~3.81-100.40~~ | ~~＜0.001~~ | ~~6.6~~ | ~~1.03-42.83~~ | ~~0.04~~ |
| ~~IAH(n)~~ | ~~3.93~~ | ~~1.67-9.22~~ | ~~0.002~~ | ~~2.8~~ | ~~1.05-7.58~~ | ~~0.04~~ |

~~OR: odds ratio; CI: confidence interval; BMI: body mass index; Scr: serum creatinine; RV: right ventricular; LV: left ventricular~~
